# Comparison of excitation energy transfer in cyanobacterial photosystem I in solution and immobilized on conducting glass

**DOI:** 10.1007/s11120-016-0312-4

**Published:** 2016-10-01

**Authors:** Sebastian Szewczyk, Wojciech Giera, Sandrine D’Haene, Rienk van Grondelle, Krzysztof Gibasiewicz

**Affiliations:** 1 Department of Physics, Adam Mickiewicz University, ul. Umultowska 85, 61-614 Poznan, Poland; 2 Department of Physics and Astronomy, Vrije Universiteit, De Boelelaan 1081, 1081 HV Amsterdam, The Netherlands

**Keywords:** Photosystem I, Time-resolved fluorescence, Streak camera, Excitation energy transfer, Cyanobacteria, Photovoltaics, Biophotovoltaics, Red chlorophylls

## Abstract

Excitation energy transfer in monomeric and trimeric forms of photosystem I (PSI) from the cyanobacterium *Synechocystis* sp. PCC 6803 in solution or immobilized on FTO conducting glass was compared using time-resolved fluorescence. Deposition of PSI on glass preserves bi-exponential excitation decay of ~4–7 and ~21–25 ps lifetimes characteristic of PSI in solution. The faster phase was assigned in part to photochemical quenching (charge separation) of excited bulk chlorophylls and in part to energy transfer from bulk to low-energy (red) chlorophylls. The slower phase was assigned to photochemical quenching of the excitation equilibrated over bulk and red chlorophylls. The main differences between dissolved and immobilized PSI (iPSI) are: (1) the average excitation decay in iPSI is about 11 ps, which is faster by a few ps than for PSI in solution due to significantly faster excitation quenching of bulk chlorophylls by charge separation (~10 ps instead of ~15 ps) accompanied by slightly weaker coupling of bulk and red chlorophylls; (2) the number of red chlorophylls in monomeric PSI increases twice—from 3 in solution to 6 after immobilization—as a result of interaction with neighboring monomers and conducting glass; despite the increased number of red chlorophylls, the excitation decay accelerates in iPSI; (3) the number of red chlorophylls in trimeric PSI is 4 (per monomer) and remains unchanged after immobilization; (4) in all the samples under study, the free energy gap between mean red (emission at ~710 nm) and mean bulk (emission at ~686 nm) emitting states of chlorophylls was estimated at a similar level of 17–27 meV. All these observations indicate that despite slight modifications, dried PSI complexes adsorbed on the FTO surface remain fully functional in terms of excitation energy transfer and primary charge separation that is particularly important in the view of photovoltaic applications of this photosystem.

## Introduction

Photosystem I (PSI) is a well-described pigment-protein complex with a quantum efficiency of photon to electron conversion near unity (Gobets and van Grondelle 2001). This promising feature combined with high stability beyond the natural lipid membrane and relatively high thermal stability (Balme et al. 2001) causes this complex to become the object of interest for biophotovoltaic applications. Many groups have investigated the properties of PSI deposited onto various substrates. The most commonly used substrates are: chemically modified gold electrodes (Ciesielski et al. 2010; Mukherjee and Khomami 2011), semiconductors like TiO_2_ or ZnO (Mershin et al. 2012; Yu et al. 2015), and graphene (Gunther et al. 2013; Feifel et al. 2015). For most of the aforementioned works, the main evidence of intactness of the structure and function of PSI was its photoelectrochemical response. However, an additional evidence of stability could be obtained by a detailed study of the first steps of energy and electron transfer processes. Therefore, we decided to investigate and compare the excitation dynamics of PSI suspended in solution and PSI deposited and immobilized onto FTO conductive glass as a substrate (immobilized PSI or iPSI).

The cyanobacterial PSI core, in contrast to plant and green algae, naturally occurs in two forms: monomeric and trimeric. Both forms coexist in vivo, and the ratio between them can be modulated, for instance by varying the salt concentration (El-Mohsnawy et al. 2010). Moreover, the PSI complex from plants and green algae in vivo is associated with an external antenna system—light harvesting complex I (LHCI) which is not present in cyanobacteria.

The 2.5 Å structure of the trimeric PSI core (Jordan et al. 2001) from *Synechococcus elongatus* reveals 12 protein subunits and 127 cofactors per each monomer. The composition of cofactors includes: 96 chlorophylls (Chls), 22 carotenoids (Cars), 2 phylloquinones, 3 iron-sulfur clusters, 4 lipids, one metal ion (presumably Ca^2+^) and 201 water molecules. Two large subunits (PsaA, PsaB) form a heterodimer; they bind most of the Chls and Cars molecules and coordinate the functionally most important part of PSI reaction center (RC), where charge separation occurs. The RC is composed of 6 Chls (two of them forming P700, two accessory Chls labeled A, and two primary electron acceptors labeled A_0_), two phylloquinones and three iron-sulfur cofactors. The remaining Chls and Cars form the core antenna whose role is to harvest photons and transfer excitation energy to the RC. The primary donor of PSI (P700) absorbs at around 700 nm, whereas the vast majority of Chls (“bulk” Chls) absorbs at around 680 nm. However, in almost all PSI complexes, a pool of Chls that absorbs at an energy lower than the primary donor can be distinguished. These forms are the so-called red or long-wavelength Chls (“red” Chls) due to their redshifted absorption. While all red Chls are present in cyanobacterial PSI core complexes, in green algae and plants most of the red Chls are located in LHCI (Croce et al. 1998; Giera et al. 2014). The number of red Chls and their spectroscopic characteristics are species-dependent (Gobets et al. 2001), and they constitute 3–10 % of the total amount of Chls. It was proposed that the unusual spectroscopic features of the red forms result from strong pigment–pigment interactions (Gobets et al. 1994; Jordan et al. 2001; Engelmann et al. 2001) which causes the mixing of excitonic and charge transfer states (Romero et al. 2008; Novoderezhkin et al. 2016).

The red Chls may arise as a result of excitonic interactions between two, three or more Chls and their subsequent mixing with charge transfer states. The possibility of the existence of such dimers and trimers was suggested based on the cyanobacterial PSI structural model (Jordan et al. 2001) and confirmed by simulations (Gobets et al. 2003).The exact location of the red forms in the cyanobacterial PSI is still under discussion. Some reports suggest that their distance from P700 is rather large (Vaitekonis et al. 2005). Others indicate a close location of red Chls to carotenoids (Elli et al. 2006). Another approach assumes that at least a part of red forms is located in the peripheral region of the PSI core. This is in agreement with the observation that the number of red Chls is affected by the aggregation state of PSI—in cyanobacterial PSI trimers, the amount of red forms is generally higher than in monomers. This effect was observed for different species of cyanobacteria: *Thermosynechococcus elongatus, Synechocystis* sp. PCC 6803 and *Arthrospira platensis* (Gobets et al. 2003; Karapetyan et al. 2014).

In *Synechocystiss* sp. PCC 6803, one red Chls pool was reported (Gobets et al. 2003). The absorption maximum of the red Chls band was around 708 nm (C708), and it was proposed to be most likely formed by three distinct excitonically coupled dimers. The estimated amount of red Chls per 100 antenna Chls was about 3 in monomeric PSI and 4–5 per monomer in trimeric PSI. However, recent research narrowed the number of coupled pigment dimers in monomeric PSI down to two (Mazor et al. 2014), but in return, excluded the possibility of the existence of excitonically coupled Chls trimers. In the literature, an alternative way of describing red Chls pools in *Synechocystis*, based on hole burning experiments, can be found (Hayes et al. 2000; Brecht and Nieder 2008). The differences in electron–phonon coupling in the blue and red region of the C708 absorption band were an argument to suggest that the C708 band observed at low temperatures can be split into two absorption bands: C706 and C714.

PSI from *Synechocystis* has earlier been investigated using picosecond time-resolved fluorescence (Hastings et al. 1994; Turconi et al. 1996; Gobets et al. 2001, 2003). Despite differences in experimental setups and sample purity, all results are similar in terms of Chls spectral distribution and excitation decay that can generally be described using a bi-exponential model (4–6 and 20–25 ps, with the addition of a non-decaying component). In the aforementioned works, the faster component is assigned to the equilibration of the excitation energy between different spectral forms of Chls, whereas the second one describes the decay of the spectrally equilibrated excited state. In addition, the overall excitation dynamics is very similar in monomeric and trimeric forms, which suggests their similarity in terms of charge separation efficiency.

## Materials and methods

The experiments were performed both for monomeric and trimeric PSI complexes from *Synechocystis* sp. PCC 6803.

### Cell growth and preparation of thylakoids

Cell cultures were grown in BG-11 medium (Rippka et al. 1979) under continuous white light and stirring in small volumes (250 ml flasks, to improve growth rate) in ambient air at 25 °C. After 7–10 days (log phase) cells were harvested by centrifugation at 2000×*g* for 15 min in a MPW-251 swing-out rotor, and resuspended in a buffer containing 30 mM Bis Tris–HCl, pH 6.5, 0.3 M mannitol, 5 mM ethylenediaminetetraacetic acid (EDTA), 10 mM NaCl, 5 mM NaH_2_PO_4_ and 5 mM K_2_HPO_4_ (buffer A). Cultures from ~8 l of such cultivation were concentrated in buffer A at a chlorophyll *a* concentration of ~1 mg/ml. The chlorophyll concentration was determined according to Porra et al. (1989). In order to lyse the cell walls, 0.2 % lysozyme was added to the sample (2 h incubation in dark at 37 °C). Next, protease inhibitors from 100 mM stocks [benzamidine, caproic acid and phenylmethylsulfonyl fluoride (PMSF)] were added to a final concentration of 1 mM each, the sample was transferred on ice, then cells were disrupted using sonication (Sonics Vibra-Cell VCX130), with “10 s on–30 s off” cycles, for 10 min. The obtained extract was centrifuged at 500×*g* for 5 min in a MPW-251 swing-out rotor, to separate the membranes from the cell debris.

### PSI extraction and purification

Phycobilisomes were removed as follows. One volume of buffer B [20 mM Bis Tris, pH 6.5, 5 mM NaH_2_PO_4_, 5 mM K_2_HPO4, 5 mM EDTA and 0.06 % n-dodecyl β-D-maltoside (β-DM)] was added to the sample and centrifuged at 235,000×*g* for 30 min at 4 °C in a Sorvall S100-AT6 rotor. The supernatant was discarded, and the procedure was repeated twice with buffer B with 0.03 % β-DM. Thylakoids were resuspended in buffer C (20 mM Bis Tris, pH 6.8, 5 mM CaCl_2_, 5 mM MgCl_2_, 10 mM NaCl, 1.5 % taurine and 0.03 % β-DM) at a chlorophyll *a* concentration of ~1 mg/ml, and 10 % β-DM was added (v/v) to a final concentration of 1 % with 1 mg/ml of chlorophyll *a*. The sample was incubated at 4 °C for 20 min in the dark, and subsequently ultra-centrifugation was performed (110,000×*g*, 15 min, 4 °C in a Sorvall S100-AT6 rotor). The final stage of isolation is based on Ramesh et al. (2004) with several modifications. The supernatant was loaded onto a self-packed DAEA Toyopearl 650 S column (weak anion exchanger), equilibrated previously with buffer C. The column was washed with buffer C containing 5 mM MgSO_4_ at a flow rate of ~5 ml/min. Next, a gradient of MgSO_4_ (5–150 mM) in buffer C was applied. The concentration of MgSO_4_ needed for elution was about 15 mM for monomeric PSI and above 50 mM for trimeric PSI. Those values, however, depend strongly on the concentration of the other salts and pH of the buffer. Fractions with a *Q*_*y*_ maximum absorption band above 678 nm were pooled and concentrated using Amicon 100 kDa ultra-filters. In order to remove excess of MgSO_4_, samples were dialyzed in buffer D (buffer C without taurine), using the aforementioned filters and kept frozen at −20 °C until further use.

### Immobilization of the PSI complexes onto FTO glass

Deposition of the PSI complexes onto PSI glass was performed as follows. Pre-diluted PSI samples (with ninefold reduced initial detergent concentration) were washed by ultracentrifugation (220,000×*g* for 30 min at 4 °C in a Sorvall S100-AT6 rotor). The initial detergent dilution was sufficient for partial precipitation of the PSI complexes. About 80 % of the supernatant was discarded. Then, the same volume of buffer without detergent was added. Aliquots of 1 ml of PSI sample were then dialyzed in distilled water (of volume exceeding the volume of the PSI solution 600 times) with addition of 10 mM Bis Tris pH 7.0.

Samples with a reduced amount of salts and detergent were deposited onto FTO glass, with the help of an externally applied electrical field. A drop of PSI solution was placed between two FTO electrodes, separated by a ~2 mm spacer. Next, the electrodes were connected to a stable voltage source (NDN DF-1730). Different voltage values, electrodes polarization and time of electrically assisted deposition were tested—the most homogeneous and durable layers were formed with 2.5 V applied for 5 min. Another group also found similar conditions as optimal (Mukherjee and Khomami 2011). PSI complexes migrated preferentially to the positive plate, which indicates that their net charge under such conditions was negative. This observation is consistent with the isoelectric point of plant PSI at pH 4.9–5.0 (Liu et al. 2012) and inconsistent with the report by Mukherjee and Khomami (2011) who observed the PSI particles migration toward the negative electrode. This discrepancy could be caused by the other type of substrate (alkanethiolate self-assembled gold electrode), different PSI complexes used (*Thermosynechococcus elongatus*) or a different ionic strength of the used buffer. The estimated number of PSI layers deposited onto FTO was about 14 assuming the cross-sectional size of a PSI monomer of the order of 100 nm^2^. OD_679 nm_ of the PSI complexes immobilized on FTO was about 0.04.

### Steady-state absorption measurements

Room-temperature absorption spectra were collected using a Hitachi-1900 UV/VIS spectrophotometer. Measured samples were suspended in buffer D and normalized in the *Q*_*y*_ band.

### Fluorescence correlation spectroscopy (FCS)

In the FCS technique, the diffusion time of a light-emitting particle through the confocal volume is measured. The diffusion time of a molecule in a particular solution is proportional to its hydrodynamic radius. The hydrodynamic radius (also called Stokes radius) is a radius of a hypothetical hard sphere, which diffuse in a particular solution at the same rate as the molecule under observation. Any solvent or detergent molecules permanently attached to the surface of the molecule under study contribute to the measured hydrodynamic radius.

Experiments were carried out using a ConfoCor2 instrument (Zeiss, Jena, Germany) equipped with a water-immersion objective Zeiss C Apochromat 40×/1.2 W. The light source was a standard Helium–Neon 633 nm 5 mW laser with an output power set to 3 %. The detection line contained a 650 nm low pass filter. The SP value (parameter specifying dimensions of the focus) was set upon measurement of the Atto633 dye in water to a value of 6.3. The samples of PSI were diluted to OD_679nm,1cm_ ~ 1 in buffer D, then diluted again 50 times with distilled water. Measurements aiming to compare the diffusion time of monomeric and trimeric PSI complexes were repeated 8 times under identical conditions, each time using a fresh sample and then averaged. To improve the signal/noise ratio of the autocorrelation function, each measurement series was based on 40 scans of the diffusion time, with a duration of 15 s each. More details about the used setup can be found in Banachowicz et al. (2014). The results obtained from the experiment were verified by simulation based on a simple bead model, performed in the Hydro++ program (Ortega et al. 2007). The simulation concerned a comparison of the diffusion time between a single spherical bead and three identical beads linked together in a triangular arrangement which is an approximation of the geometry known for trimeric PSI (Jordan et al. 2001; Germano et al. 2002).

### Time-resolved fluorescence spectroscopy

For time-resolved fluorescence spectroscopy measurements, the PSI samples in solution were diluted in buffer D to OD_679nm,1cm_ ~ 1. Both the monomeric and trimeric forms of PSI were measured in two cases: a) P700 in the neutral state (open RC)—addition of 20 µM phenazine methasulfate (PMS) supplemented with 20 mM ascorbic acid; b) P700 in the oxidized state (closed RC)—addition of 3 mM potassium ferricyanide. Examples of using those agents to modulate the RC state can be found in the literature (Byrdin et al. 2000; Gobets et al. 2001; Giera et al. 2014). Before deposition of PSI complexes onto FTO, they were diluted in buffer D without addition of any reducing or oxidizing agents.

The time-resolved fluorescence spectroscopy experiments were carried out with a streak camera setup (Laser Center, Vrije Universiteit, Amsterdam) at room temperature. All measurements were performed using 400-nm, 100-fs laser pulses, repetition rate 125 kHz for excitation. Those pulses were generated in an 800 nm titanium:sapphire laser (Coherent Vitesse) amplified by a regenerative amplifier (Coherent RegA 9000) and fed into an optical parametric amplifier (Coherent OPA 9400) where the second harmonic was generated.

In order to avoid excessive irradiation, solutions of PSI were placed in a rotating cuvette and FTO glasses with adsorbed iPSI and were moved by a mechanical motion controller. The energy of a single pulse was below 1 nJ at the spot size of approximately 150 µm. At this excitation level, effects due to singlet–singlet annihilation could not be observed.

The fluorescence was collected at a right angle with respect to the excitation beam. Spectral and time resolution was achieved by the spectrograph (Chromex 250IS) and the streak camera (Hamamatsu C5680), respectively. The streak images were recorded by Hamamatsu C4880 CCD device, in three different time domains (time ranges, TR): TR1 ~140 ps, TR2 ~400 ps and TR3 ~1400 ps. Time resolution in those modes was ~3.5, ~6 and ~15 ps, respectively. The wavelength scale of the spectrograph was calibrated using an argon lamp.

The exposure time per image was chosen so as to obtain a similar signal for each time range and each sample (about 4000 counts at maximum). In order to improve the signal/noise ratio, four images of each sample were collected and averaged. Each experiment was corrected by background subtraction and then analyzed with Glotaran software (Snellenburg et al. 2012) using global and target analysis. It was previously demonstrated how the excitation dynamics in PSI can be described using those two analysis approaches (Giera et al. 2010, 2014). Mathematical principles of these approaches can be found in van Stokkum et al. (2004).

## Results

### Size of monomeric and trimeric PSI particles

**Fig. 1:**
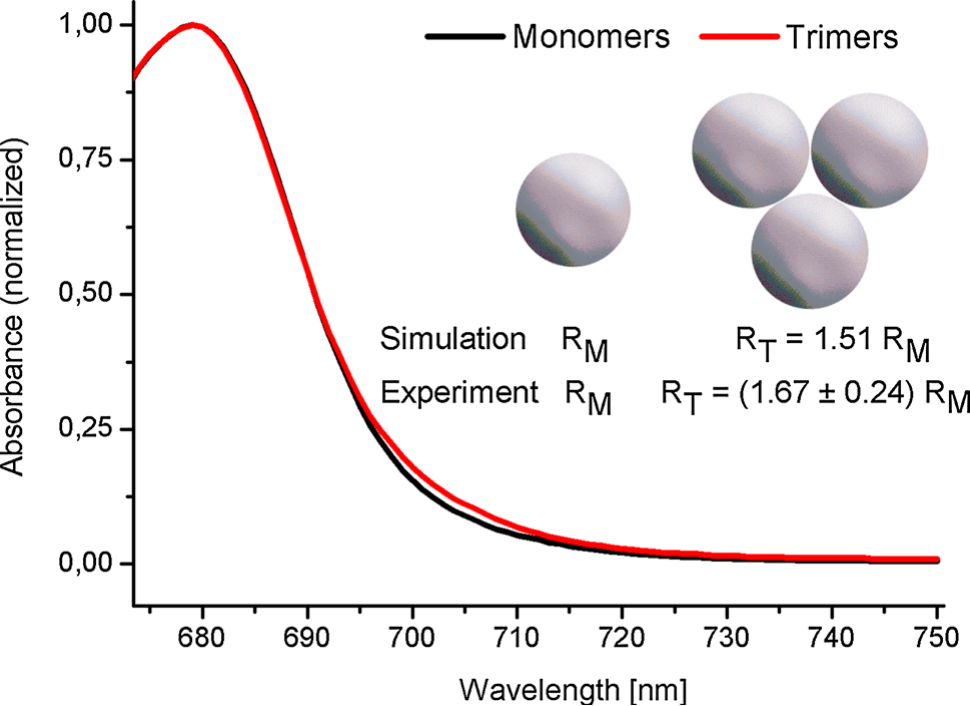
Comparison of room-temperature absorption spectra of monomeric (*black*) and trimeric (*red*) PSI complexes in the *Q*_*y*_ band region (normalized at the *Q*_*y*_ band’s maximum). *Inset* bead models of monomeric and trimeric PSI complexes and values of hydrodynamic radii obtained for trimeric forms (*R*_T_) relative to monomeric forms (*R*_M_) either in simulations or in fluorescence correlation spectroscopy experiments

Standard isolation procedures were applied in order to obtain monomeric and trimeric PSI complexes (see Materials and methods). In order to further confirm the homogeneity of the two fractions, fluorescence correlation spectroscopy was applied. This technique allows the estimation of diffusion coefficients of dissolved emitting molecules and their effective (hydrodynamic) radii (assuming a spherical shape of the molecules). As shown in the inset to Fig. 1, the hydrodynamic radius of the trimeric PSI was about 1.67 ± 0.24 times larger than that of the monomeric PSI. This number is in a very good agreement with a ratio of 1.51 theoretically simulated for a trimer of three spherical beads arranged in a way shown in the inset of Fig. 1. Thus, we concluded that the fractions of monomeric and trimeric PSI were highly homogenous. The absolute value of the hydrodynamic radius of the trimeric PSI was 11.7 ± 1.7 nm which is comparable to the values reported previously (~9.5–10.5 nm radius, ~9 nm height), using electron microscopy (EM) and X-ray diffraction techniques (Kruip et al. 1997; Schubert et al. 1997). Obviously, the differences come from the rough approximation of the PSI particle (sphere vs. disk-like particle). Moreover, in the FCS technique it is not possible to perform the correction for attached detergent (which causes an artificial increase of the hydrodynamic radius). However, a comparison of obtained trimeric PSI dimensions from EM and X-ray diffraction experiments shows a contribution of the detergent layer of about 0.7 nm (Germano et al. 2002). On the other hand, detergent contribution of 1.7 nm was estimated on the basis of EM studies on plant PSI complexes (Boekema et al. 1990).

Figure 1 compares the shapes of the long-wavelength slopes of the *Q*_*y*_ absorption bands in monomeric and trimeric PSI. Trimeric complexes show larger absorption at 705 ± 10 nm than monomeric complexes thus demonstrating the existence of extra red Chls in the former preparation. This effect was reported previously, and the extra red Chls were proposed to be formed at the interface of interacting PSI monomers forming a trimer (Gobets et al. 2003; Karapetyan et al. 2014).

### Time-resolved fluorescence for PSI in solution: global analysis

**Fig. 2:**
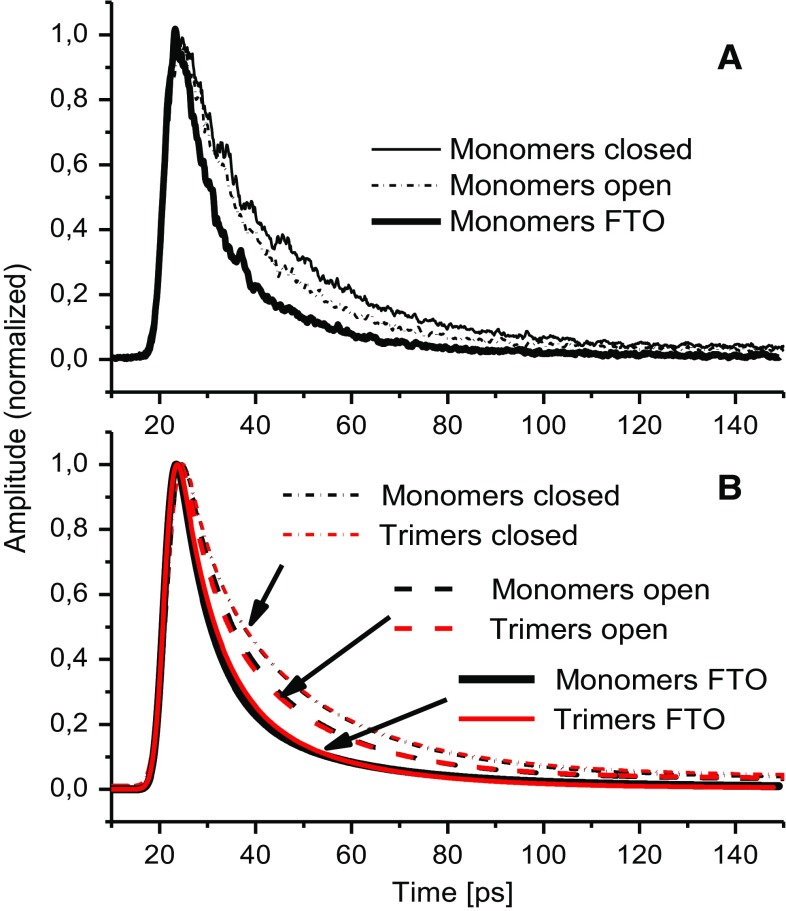
Fluorescence decay traces measured at 686 nm following excitation at 400 nm. **a** Raw data measured for monomeric PSI complexes in solution (*open* or *closed*) or attached to FTO. **b** Fits to the data measured for monomeric and trimeric PSI complexes in solution or attached to FTO

**Fig. 3:**
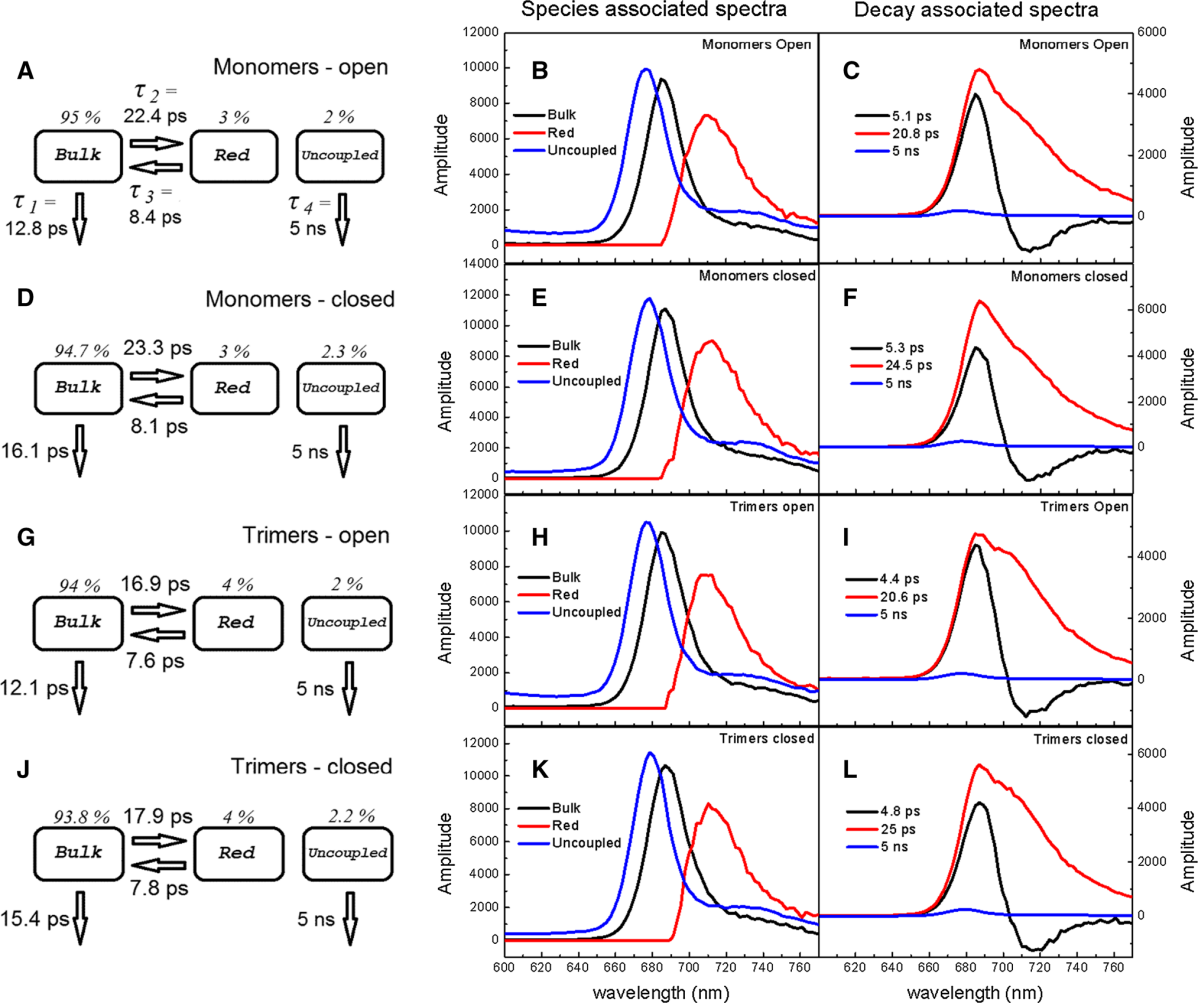
Fluorescence decay analysis of monomeric and trimeric PSI complexes in solution being either in open or in closed state. Excitation wavelength was 400 nm. The *left-most panels* (**a**, **d**, **g**, **j**) present common compartmental model underlying target analysis, including estimated molecular lifetimes and initial populations of particular compartments; the *central panels* (**b**, **e**, **h**, **k**) results of the target analysis (species-associated spectra); the *right-most panels* (**c**, **f**, **i**, **l**) results of the global analysis (decay-associated spectra). The uncertainty of molecular lifetimes was estimated at ±0.5 ps

Figure 2a presents fluorescence decay kinetics at 686 nm (maximum of the room temperature fluorescence emission spectrum) of monomeric PSI in solution (either in open or in closed state) as well as monomeric PSI immobilized on FTO conducting glass. One can see that permanent oxidation of the primary donor (closed state) leads to a deceleration of the fluorescence decay. The average fluorescence lifetime decay, *τ*_av_, increases from 13.8 to 16.7 ps (Table 1). Interestingly, these two values are almost identical to those found for algal PSI core in the open and closed state, respectively (Giera et al. 2014). On the other hand, immobilization of probably mostly closed monomeric PSI on FTO (see below) leads to a significant acceleration of the decay (*τ*_av_ = 10.6 ps). The same effects were observed for trimeric PSI (Fig. 2b; Table 1). Importantly, formation of trimers gives rise to only small differences in kinetics, much smaller than the effects caused by primary donor oxidation or PSI immobilization on FTO. Figure 3c, f, i, l shows the results of global analysis of the fluorescence decays in a wide spectral range from 600 to 770 nm for monomeric and trimeric PSI in the open and closed state. In each case, the fit was performed using three exponential functions; addition of another exponential component did not improve the fit quality significantly. One can see that in all cases, the decay is dominated by a ~4–5 and ~21–25 ps components (decay-associated spectra or DASes) and that the third component with a 5-ns lifetime is of very small amplitude. The spectral shape of the fastest component is characterized by a larger positive band with maximum at ~685 nm and a smaller negative band with a minimum at ~714 nm which clearly indicates coexistence of two processes occurring on the same time scale: (1) excitation energy transfer from bulk to red Chls and (2) excitation quenching assigned to charge separation in the reaction center (photochemical quenching). This conclusion concerning the latter process is based on the fact that the positive band is larger than the negative one (Gobets et al. 2001). The second ~21–25 ps component with a maximum at ~687 nm was attributed to photochemical quenching of the remaining excitation equilibrated over bulk and red Chls. Only one pool of red Chls was resolved in line with previous reports for *Synechocystis* sp. PCC 6803 (Gobets et al. 2001; Gobets and van Grondelle 2001). The DAS associated with the lifetime of ~5 ns peaks at 677 nm, to the blue from the bulk Chls that is characteristic for Chls uncoupled from PSI RCs. The nature of uncoupled Chls is not clear although its ~10 nm blue shift relative to bulk Chls indicates specific interaction with the environment different from that in fully functional PSI complexes. In the following, we will focus only on the two faster components.

**Table 1:** Parameters estimated from fluorescence decay of PSI complexes

Sample	Average lifetime @686 nm *τ*_av_ (ps)	SAS band maximum (nm)		Δ*λ* (nm) *δ* = ± 0.5 nm	Δ*H* ^0^ (meV) *δ* = ± 2.6 meV	*τ*_1_ (ps)	*τ*_2_ (ps)	*τ*_3_ (ps)	*τ*_4_ (ps)	Δ*G* ^0^ (meV) *δ* = ± 1 meV	*N*_*r*_^eff^
		Bulk *λ*_*b*_	Red *λ*_*r*_								
Monomers open	13.8 (13.8)^$^	685.5	709.5	24	61	12.8 (18)* (8.9)^#^	22.4 (31)*	8.4 (8.6)*	5000 (4800)*	−25 (−33)*	3.4 ± 0.5
Monomers closed	16.7 (17.4)^$^	686.5	711	24.5	62	16.1 (11.4)^#^	23.3	8.1	5000	−27	3.0 ± 0.5
Trimers open	12.8	685.5	709	23.5	60	12.1 (18)*	16.9 (18)*	7.6 (8.9)*	5000 (6700)*	−21 (−18)*	4.2 ± 0.7
Trimers closed	16.4	687.5	711.5	24	61	15.4 (15.6)^&^	17.9 (20.8)^&^	7.8 (7.1)^&^	5000	−22 (−27)^&^	3.9 ± 0.6
Monomers FTO	10.6	688	708.5	20.5	52	10.3	30	15.4	228	−17	6.3 ± 1
Trimers FTO	11.4	687.5	710.5	23	59	10.5	36.7	14.1	442	−25	3.9 ± 0.5

Average fluorescence lifetime, *τ*_av_, was calculated from the equation: *τ*_av_ = (*τ*_1_ *A*_1_ + *τ*_2_ *A*_2_ *)*/*(A*_1_ + *A*_2_ *),* where *τ*_1_ and *τ*_2_ are lifetimes, and *A*_1_ and *A*_2_ are the amplitudes (at 686 nm) of the two faster components obtained from the global analysis (Figs. 3, 5). Band’s maxima were read out from the respective SASes (Figs. 3, 5), and molecular lifetimes, *τ*_*i*_, defined in Fig. 3a, were rewritten from Figs. 3 and 5. In the brackets, there are molecular lifetimes reported in Gobets et al. (2001; brackets with symbol “*”), in Giera et al. (2010; with symbol “#”; algal PSI core) and in van Stokkum et al. (2013; with symbol “&”) as well as average lifetimes reported for algal PSI core in Giera et al. (2014; with symbol “$”). Δ*λ* is a difference between the maxima of red and bulk Chls SASes. Enthalpy difference (Δ*H* ^0^), free energy difference (Δ*G* ^0^) and effective number of red chlorophylls (*N*_*r*_^eff^) were calculated according to Eqs. 3, 5, 6 and 8. The uncertainty of molecular lifetimes necessary to estimate *δ*Δ*G* ^0^ was taken ±0.5 ps

The respective DASes obtained for monomeric and trimeric PSI either in open or in closed states are very similar to each other in terms of lifetimes and spectral shapes (compare Fig. 3c–i, and Fig. 3f–l). On the other hand, when comparing open and closed PSI, either for monomeric or trimeric PSI, one can see that the lifetime of the fastest decay phase insignificantly increases upon RC closure (from 5.1 to 5.3 ps for monomers and from 4.4 to 4.8 ps for trimers), whereas the slower decay phase increases significantly from ~21 to ~25 ps for both preparations. Moreover, the relative amplitudes of the two phases change—upon RC closure the amplitudes of the slower DASes increase relative to those of the fast DASes (compare Fig. 3c–f, and I–l). All these changes contribute to a slowing down of the fluorescence decay after chemical oxidation of P to P^+^ (compare Fig. 2).

**Fig. 4:**
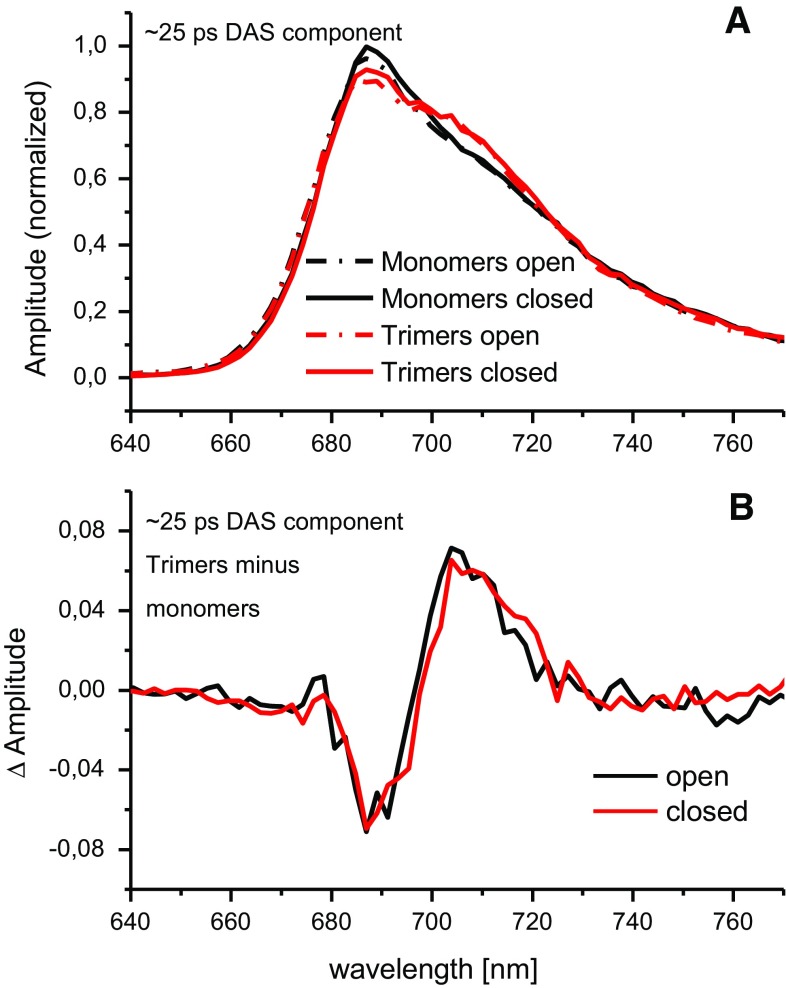
Comparison of the ~21–25 ps DAS component associated with monomeric and trimeric PSI complexes being either in open or in closed state. **a** Spectra were normalized to the same area under the curves. **b** Trimer minus monomer spectra were calculated both in open and in closed states

Another interesting point is the difference between the spectral shapes of the slower DAS in the case of monomeric and trimeric PSI. The most prominent difference is a “bump” on the long-wavelength slope of this DAS clearly seen in trimers and absent in monomers (compare Fig. 3c, f to l, l). The respective spectra were normalized to the same integrals in Fig. 4a and then subtracted from one another. Results of these subtractions are shown in Fig. 4b. As seen, the state of the RC (open or closed) does not influence the shape of the respective DASes, only the formation of trimers does. Maxima of the differential spectra peak at ~705 nm (Fig. 4b), which is a similar wavelength as that for which the difference between absorption spectra of trimeric and monomeric PSI is maximal (compare to Fig. 1). Thus, we propose that the extra absorption seen in trimeric PSI (Fig. 1) and the “bump” seen in fluorescence DASes (Fig. 3i, l) have the same origin: they are due to the extra red Chls present only in trimeric PSI.

### Time-resolved fluorescence for PSI in solution: target analysis

In order to obtain spectra of bulk and red Chls as well as to estimate molecular rate constants or their reciprocals—molecular lifetimes—we performed target analysis for monomeric and trimeric PSI in solution. We tested a few compartmental models including those with reversible primary charge separation in the RC. The simplest model, fitting very well the raw data from monomeric and trimeric PSI both in open and closed state, was composed of three compartments: bulk, red and uncoupled Chls (Fig. 3a, f, g, j). Addition of more compartments, for example, a reversible charge separated state in the RC, improved the fit quality insignificantly and yielded physically uninterpretable results. We assumed that the compartment “Uncoupled” Chls is unconnected from compartments “Bulk” and “Red.” This assumption is justified by the long 5-ns lifetime characteristic for unquenched Chls in solution. In line with the small amplitudes and maximum wavelength of the 5-ns DASes (Fig. 3c, f, i, l), the target analysis yielded only ~2 % contribution and a reasonable spectrum (SAS, species-associated spectrum) of uncoupled Chls for all four samples (Fig. 3a, d, g, j and b, e, h, k). In order to achieve the bi-exponential fluorescence decay from the two remaining compartments as observed in global analysis (Fig. 3c, f, i, l), it was necessary to introduce reversibility of excitation energy transfer between bulk and red Chls. Thus, the faster ~4–5-ps component is due to two competing processes—bulk Chls excitation trapping (charge separation; depicted by lifetime *τ*_1_ in Fig. 3a) and excitation energy transfer from bulk to red Chls (depicted by lifetime *τ*_2_ in Fig. 3a)—whereas the slower ~21–25 ps component is due to photochemical quenching of excitation equilibrated over bulk and red Chls and is additionally dependent on lifetime *τ*_3_ (Fig. 3a).

Molecular lifetimes obtained from the target analysis for open PSI monomers are shown in Fig. 3a. Irreversible charge separation is depicted by the lifetime *τ*_1_ = 12.8 ps, whereas the pool of red Chls is populated with lifetime *τ*_2_ = 22.4 ps. The effective back energy transfer from red to bulk Chls equals *τ*_3_ = 8.4 ps and is almost three times faster than the forward energy transfer. The initial distribution of excitation between bulk and red Chls, 94.7:3 %, was set according to the estimation that 3 red Chls are present in one PSI monomer in solution (see below). However, the exact value of this initial distribution was not critical for the obtained results of the target analysis, meaning that for different initial distributions the fitting always converged to the same final values of molecular rate constants and the same shapes of SAS. The maximum of the bulk Chls SAS amplitude is somewhat higher than that of the red Chls SAS, but the width of the latter spectrum is larger than that of the former one (Fig. 3b). Thus, the relative areas under the bulk and red Chls SAS are similar to one another. The maximum of red Chls SAS is red shifted relative to that of bulk Chls by 24 nm from ~685.5 to ~709.5 nm (Fig. 3b; Table 1).

Closing of monomeric PSI RCs did not lead to significant changes in lifetimes *τ*_2_ and *τ*_3_, but the quenching lifetime *τ*_1_ increased significantly by ~25 % from 12.8 to 16.1 ps upon RC closure (Fig. 3d). These effects were expected since permanent oxidation of the primary donor should not influence coupling between bulk and red Chls and the quenching properties of PSI were reported to be weakened in a similar way following RCs closure for *Chlamydomonas reinhardtii* (Giera et al. 2010, 2014). Thus, we conclude that the only reason for the slower fluorescence decay in closed PSI monomers (Fig. 2) is the slower quenching by closed RCs. The shapes and positions of the bulk and red Chls SASes of open and closed PSI RCs were similar.

Molecular lifetimes and SASes obtained from the target analysis for open PSI trimers shown in Fig. 3g, h are in many aspects similar to the respective lifetimes and SASes discussed above for PSI monomers (Fig. 3d, e). The only significant difference is the faster forward excitation energy transfer from bulk to red chlorophylls: the lifetime *τ*_2_ = 16.9 ps is smaller by ~25 % as compared with the respective lifetime for monomers (22.4 ps). However, this difference does not strongly influence the overall fluorescence kinetics. The reason for the accelerated bulk to red Chls energy transfer is most likely the presence of extra red Chls in trimeric PSI discussed above. One needs to remember that the “Red” compartment in our model reflects a “mean” red Chls pool, most likely composed of a few individual red Chl-states, each of them having a potentially different spectrum and coupling to bulk Chls.

Closing trimeric PSI leads to only one significant change: ~25 % increase of *τ*_1_ from 12.1 to 15.4 ps, very similar to that observed for monomeric PSI.

### Time-resolved fluorescence for immobilized PSI: global and target analysis

**Fig. 5:**
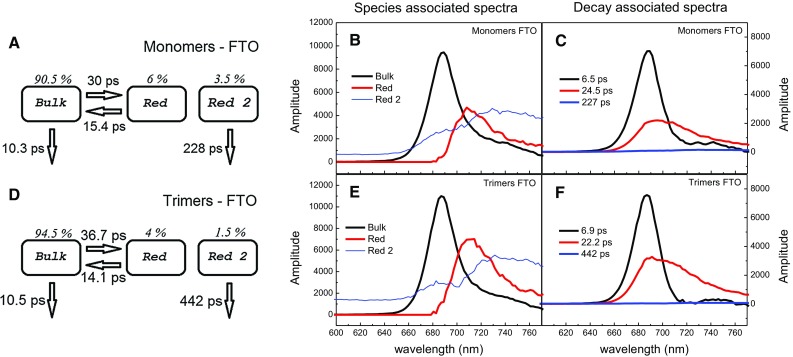
Fluorescence decay analysis of monomeric and trimeric PSI complexes immobilized onto FTO. Excitation wavelength was 400 nm. The *left-most panels* (**a**, **d**) present common compartmental model underlying target analysis, including estimated lifetimes and initial populations of particular compartments, the *central ones* (**b**, **e**) results of the target analysis (species-associated spectra) and the *right-most panels* (**c**, **f**) results of the global analysis (decay-associated spectra)

The redox state of the primary donor in dried PSI monomers and trimers immobilized on FTO (iPSI) is uncertain. However, regarding that no chemicals intended to keep RCs open or closed were added to the PSI solution used for their adsorption on FTO and that under such conditions PSI complexes in solution stay preferentially in the closed state (data not shown), we assume that our iPSI is mostly in the closed state. Decay-associated spectra for iPSI monomers and trimers (Fig. 5c, f) are markedly different from those obtained for the respective samples in solution (Fig. 3f, l). Only the bi-exponential character of the fluorescence decay is preserved but with a faster component somewhat slower than in solution (6.5 and 6.9 ps instead of 5.3 and 4.8 ps for closed monomers and trimers, respectively) and with the slower component the same for monomers (24.5 ps) and a little bit faster for the iPSI trimers (22.3 instead 25 ps). Both in the case of iPSI monomers and iPSI trimers, the ~6–7 ps DASes, unlike the respective DASes of PSI in solution, have got only positive bands peaking at 687–688 nm, and they are missing the negative bands in the red Chls region. This may be caused by a higher contribution of excitation quenching and, thus, a lower contribution of excitation energy transfer from bulk to red Chls in this DAS, as was confirmed by the target analysis (see below). The slower, ~22–25 ps DAS both in monomers and trimers is asymmetrically broadened toward the red compared to the fast DASes. These features reveal that this phase describes quenching of excitation equilibrated over bulk and red Chls, similarly as it was in the case of PSI in solution. However, unlike in the case of PSI in solution, the relative amplitudes of the fast phase are generally much larger than those of the slower phase. This reflects a significantly accelerated fluorescence decay in iPSI shown in Fig. 2.

The third, slowest component is of very small amplitude, as in the case of PSI in solution, but its lifetime is much shorter (227 and 442 ps for monomers and trimers, respectively, compared to 5 ns in solution) and its values are highly uncertain due to small amplitudes. Additionally, the respective DASes of this component display very broad spectra slightly peaking at 730 nm (compare respective SASes in Fig. 5b, e). Apparently, these small components are due to minor fractions of extra red Chls absent for PSI in solution, likely originating from the interaction of PSI complexes with FTO or other immobilized PSI complexes. On the other hand, the fractions of uncoupled Chls (with ~5 ns lifetimes and emission peaking at 677 nm) disappear after immobilization. Similar contributions of uncoupled Chls in PSI in solution and of extra red and long-lived (~200–400 ps) Chls in iPSI may suggest that the same Chls constitute these two fractions but they undergo transition from extremely blue to extremely red forms after immobilization and drying.

Despite the markedly different fluorescence kinetics and DASes for iPSI and PSI in solution, it was possible to apply essentially the same compartmental model in a target analysis for both these types of samples. The only small difference was the replacement of the “Uncoupled” Chls compartment (Fig. 3) by an additional red Chls compartment, “Red 2” (Fig. 5a, d). Resulting molecular lifetimes for iPSI monomers and trimers show characteristic differences compared to the respective PSI complexes in solution, but the SASes associated with bulk and red Chls are again very much similar for iPSI and PSI in solution including their rough position and shapes (compare Fig. 5b, e to Fig. 3e, k). Only the relative amplitudes of red Chls SASes (“Red”) are a bit smaller in iPSI than in PSI in solution, especially for monomers (Fig. 5b). However, this may be caused by decreased oscillator strength of additional red Chls present in monomeric iPSI but not in monomeric PSI in solution (see below).

Analysis of molecular lifetimes estimated from the target analysis reveals that immobilization of both monomeric and trimeric PSI on FTO (Fig. 5a, d) leads to similar changes compared to PSI in solution. First, the quenching lifetime *τ*_1_ is shortened to 10.3–10.5 ps. These are significantly smaller values not only than those for the closed PSI in solution (15.4–16.1 ps; Fig. 3d, j), but even than those for the open PSI in solution (12.1–12.8 ps; Fig. 3a, g). Secondly, both forward and backward excitation energy transfer lifetimes between bulk and red Chls are significantly longer (*τ*_2_ = 30 ps and *τ*_3_ = 15.4 ps for monomeric iPSI, Fig. 5a; *τ*_2_ = 36.7 ps and *τ*_3_ = 14.1 ps for trimeric iPSI, Fig. 5d) than in the case of PSI in solution [*τ*_2_ = (16.9–23.3) ps and *τ*_3_ = (7.6–8.4) ps Fig. 3a,d, g, j]. This difference reveals that in monomeric and trimeric iPSI, the coupling between bulk and red Chls is weaker than for PSI in solution.

### Estimation of the enthalpy and free energy differences between bulk and red Chls: estimation of the number of red Chls

In this chapter, we apply the basic thermodynamic equations in order to estimate the “effective” numbers of bulk and red Chls present in PSI monomeric and trimeric complexes in solution and immobilized on FTO. The primary goal of these considerations is not to determine the thermodynamic functions of PSI antenna in the rigorously correct way but only to demonstrate in a quantitative way how the immobilization of PSI complexes influences the “effective” number of the red states. The “effective” numbers are the hypothetical numbers of isoenergetic bulk states and isoenergetic red states which would give the ratio of energy transfer rates from bulk to red and from red to bulk states, that is observed in the experiment (in target analysis). Thus, our main simplifying assumption is isoenergeticity of all bulk Chls and all red Chls. Similar simplification (Snellenburg et al. 2013; van Stokkum et al. 2013; Jennings and Zucchelli 2014; Le Quiniou et al. 2015) and similar way of estimation of the “effective” numbers of bulk and red Chls (Snellenburg et al. 2013; van Stokkum et al. 2013; Le Quiniou et al. 2015) were applied also in previous works. In the following, by using the term “number of (bulk or red) Chls” we mean “effective number of (bulk or red) Chls” or “number of (bulk or red) states.”

In previous sections, we showed that excitation dynamics in properly coupled PSI antenna can be successfully modeled using two emitting states: bulk and red. If transitions between these two states were the only possible reactions, then the equilibrium distribution of the excitation between bulk and red states would be described by the Boltzmann equation:

1 $$\frac{{\left[ {\text{bulk}} \right]^{\text{eq}} }}{{\left[ {\text{red}} \right]^{\text{eq}} }} = \exp \left[ { - \frac{{\Delta G^{0} }}{{k_{B} T}}} \right] = K,$$

where *k*_*B*_ is the Boltzmann constant, *T* is the absolute temperature, Δ*G* ^0^ is the standard (Gibbs) free energy difference between bulk and red states ($\Delta G^{0} = G_{b}^{0} - G_{r}^{0}$). The above equation defines also the equilibrium constant *K* for the reaction: *red* ⇆ *bulk.* The equilibrium constant *K* can be also expressed by the ratio of the rate constants for the forward (*red* → *bulk*) and backward (*bulk* → *red*) transitions, *k*_*r*→*b*_ and *k*_*b*→*r*_, in the following way:

2 $$K = \frac{{k_{r \to b} }}{{k_{b \to r} }} = \frac{{\tau_{2} }}{{\tau_{3} }}.$$

The second equality follows from the fact that the inverse of rate constants *k*_*r*→*b*_ and *k*_*b*→*r*_ is equivalent to transition times *τ*_3_ and *τ*_2_ (respectively) found in the target analysis. Thus, combining Eqs. (1) and (2) we get the equation that allows to estimate the standard free energy difference between bulk and red states based on the transition times obtained experimentally:

3 $$\Delta G^{0} = - k_{B} T\ln \left( {\frac{{\tau_{2} }}{{\tau_{3} }}} \right).$$

According to the definition of (Gibbs) free energy ($G = H - TS$), the standard free energy difference between bulk and red states can be expressed as follows:

4 $$\Delta G^{0} = \Delta H^{0} - T\Delta S^{0} ,$$

where Δ*H* ^0^ and Δ*S* ^0^ are differences in the standard enthalpy and the standard entropy (respectively) between bulk and red states ($\Delta H^{0} = H_{b}^{0} - H_{r}^{0}$ and $\Delta S^{0} = S_{b}^{0} - S_{r}^{0}$). The standard enthalpy *H* ^0^ corresponds to the electronic energy levels of the Chls’ excited states. Therefore, the standard enthalpy difference between bulk and red states can be estimated based on the fluorescence maxima of the respective SASes obtained in target analysis:

5 $$\Delta H^{0} = hc\left( {\frac{1}{{\lambda_{b} }} - \frac{1}{{\lambda_{r} }}} \right),$$

where *λ*_*b*_ and *λ*_*r*_ are the wavelengths for which SASes of bulk and red Chls, respectively, have got their emission maximum, *h* is the Planck constant, and *c* is the speed of light in vacuum.

The standard entropy difference between bulk and red states (Δ*S* ^0^) is nonzero because each of the states can be realized in a different number of ways (so-called *microstates*) determined by the number of Chls assigned to each of the two pools. In fact, the number of microstates (*N*) may be somewhat different from the exact numbers of the bulk and red Chls, because the redshift of the red Chls is assumed to be an effect of excitonic coupling within dimers or trimers of Chls mixed with charge transfer states and these two phenomena exert an unknown effect on the oscillator strength of the red Chls emitting states. Therefore, in further discussion we will relate number of microstates to the effective numbers of bulk and red Chls, *N*_*b*_^eff^ and *N*_*r*_^eff^, with the normalization condition:

6 $$N_{b}^{\text{eff}} + N_{r}^{\text{eff}} = 100$$

If we assume that all microstates within the particular state (bulk or red) are equally probable, then using the statistical definition of entropy ($S = k_{B} \ln N$) we can write the formula for the standard entropy difference between bulk and red states (Δ*S* ^0^):

7 $$\Delta S^{0} = k_{B} \left( {\ln N_{b}^{\text{eff}} - \ln N_{r}^{\text{eff}} } \right).$$

Substituting this expression to the Eq. (4) we obtain:

8 $$\Delta G^{0} =\Delta H^{0} - k_{B} T\ln \frac{{N_{b}^{\text{eff}} }}{{N_{r}^{\text{eff}} }}.$$

Therefore, having calculated Δ*H* ^0^ and Δ*G* ^0^ based on the experimental data (according to Eqs. 3 and 5, respectively) and approximating the total effective number of all Chls per PSI monomer by the number of 100 (Eq. 6) it is straightforward to calculate the effective number of red Chls per PSI monomer from the above relationship between enthalpy and free energy differences.

**Fig. 6:**
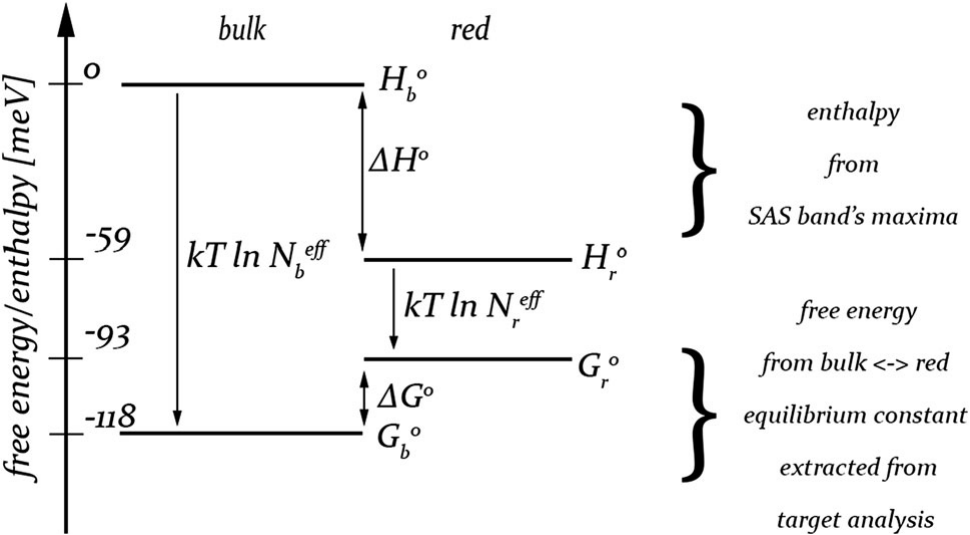
Working energetic model allowing estimation of the effective number of red chlorophylls in all types of samples (from Eqs. 3 and 4). The values of enthalpy and free energy differences as well as effective number of red Chls were taken from Table 1 for PSI trimers immobilized onto FTO. The enthalpy of a single bulk Chl molecule was arbitrarily set to 0

Target analysis showed that *τ*_2_ > *τ*_3_ for all the samples (see Table 1). Based on the Eq. 3, we can conclude that Δ*G* ^0^ < 0 for all the samples and transition from bulk to red state is effectively an uphill process. This is related to the number of bulk Chls in a monomer (*N*_*b*_^eff^) that is significantly exceeding the number of red Chls (*N*_*r*_^eff^). For a better understanding of the problem, the working energetic model for bulk and red states is presented in Fig. 6 (with the values calculated for iPSI trimers). Constructing this scheme, we used the relations arising directly from the Eq. 8:

9 $$\begin{aligned} & G_{b}^{0} = H_{b}^{0} - k_{B} T\ln N_{b}^{\text{eff}} \\ & G_{r}^{0} = H_{r}^{0} - k_{B} T\ln N_{r}^{\text{eff}} . \\ \end{aligned}$$

In order to calculate the enthalpy difference between bulk and red Chls, Δ*H* ^0^, the wavelengths of the fluorescence maxima of the respective SASes (Figs. 3, 5) were read out separately for all the samples and are collected in Table 1, as well as are the differences Δ*λ* = *λ*_*r*_ − *λ*_*b*_. Taking an experimental error of Δ*λ*, δΔ*λ* = ±0.5 nm, it was possible to estimate the experimental error of Δ*H* ^0^, δΔ*H* ^0^ = 2.6 meV. Thus, for most samples the enthalpy difference remains the same, about 60 meV. Only for monomeric iPSI, the enthalpy difference is smaller and equals 52 meV. This is the result of particularly redshifted bulk Chls (maximum at 688 nm) and particularly blueshifted red Chls (maximum at 708.5 nm), the latter being likely related to the set of specific red Chls in these samples (see below). Obviously, the enthalpy of the bulk Chls is always higher than the enthalpy of the red Chls.

Effective numbers of red Chls, *N*_*r*_^eff^, were calculated from Eqs. 8 and 6 for all samples and are collected in Table 1. The approximate numbers of (effective) red Chls for PSI in solution are 3 per monomer for monomeric PSI and 4 per monomer for trimeric PSI. It is interesting to note that according to the calculations, PSI trimers have got 1 red Chl molecule per monomer more than monomeric PSI. This observation is a quantitative interpretation of the qualitative observations of stronger absorption (Fig. 1) and fluorescence (Fig. 4) observed in trimers in the ~705 nm spectral region (see also Gobets et al. 2001). Immobilization of PSI trimers does not influence the number of red Chls. However, the effective number of red Chls is doubled and increases from 3 to 6, after immobilization and drying of monomeric PSI on FTO. This observation indicates that interactions between Chls in monomeric PSI complexes are more susceptible to modifications after deposition on the substrate than in trimeric PSI.

## Discussion

The fluorescence lifetimes and the shapes of DASes and SASes obtained for open monomeric and trimeric PSI in solution (Fig. 3) are comparable to those published previously by Gobets et al. (2001). However, the molecular lifetimes reported in the cited work (Gobets et al. 2001; see the values in brackets in Table 1) are generally larger and thus reveal somewhat slower fluorescence decay in both monomeric and trimeric complexes than in our study. In particular, photochemical quenching by charge separation (*τ*_1_) was estimated by Gobets et al. at 18 ps for both complexes which is significantly larger value than our estimates (12.1–12.8 ps). The difference may originate from partially closed RCs in the cited paper and/or somewhat different preparation. Also the energy transfer from bulk to red Chls (*τ*_2_) in monomers with open RCs was found by Gobets et al. to be significantly slower than in the case of our modeling (31 vs. 22.4 ps). Obviously, this results in larger free energy gap between bulk and red Chls reported by Gobets for monomers (open RC) compared to our case (−33 vs. −25 meV, respectively; see Table 1). The free energy gap obtained by Gobets and coworkers for trimers (open RCs) is closer to our value (−18 vs. −21 meV, respectively; see Table 1). Despite these differences, the same conclusion may be drawn based on our results and the data presented by Gobets and coworkers: namely that a smaller free energy gap exists between bulk and red Chls in trimers than in monomers, that is consistent with our observation of a larger number of red Chls in trimers.

It is worth noting that there is a very good agreement between the molecular lifetimes modeled by us and those presented in the work of van Stokkum et al. (2013; see the values in brackets in Table 1) for trimers of *Synechocystis* sp. PCC 6803. The authors do not precise if they controlled the redox state of the RCs; thus, we assumed that the RCs remained rather in closed state during their experiment. The only difference between our results is the rate of energy transfer from bulk to red Chls (*τ*_2_), which in the case of van Stokkum et al. (2013) is slightly slower than in our case (20.8 vs. 17.9 ps, respectively), that results in greater free energy gap (−27 meV). For comparison, such a free energy gap was found by us for monomers (closed RCs) rather than for trimers (closed RCs).

The effects of the PSI RC closure on fluorescence kinetics and molecular lifetimes have not been studied in detail for the cyanobacterial PSI, but similar studies have been published for PSI cores from green alga *Chlamydomonas reinhardtii* (*C. reinhardtii*) (Giera et al. 2010, 2014). In the case of algal PSI, closing of RCs caused a similar small slowing down of the fluorescence decay with molecular lifetime of photochemical quenching (*τ*_1_) increasing from 8.9 ps in open state to 11.4 ps in the closed state (Giera et al. 2010; compared to an increase from 12.8 to 16.1 ps in cyanobacterial PSI monomers; Table 1) and average fluorescence lifetime, estimated across whole spectra, increasing from 13.8 to 17.4 ps (Giera et al. 2014; compared to an increase from 13.8 to 16.7 ps measured at 686 nm in cyanobacterial PSI monomers; Table 1). The almost perfect agreement of the average lifetimes and much shorter molecular lifetimes in the case of algal PSI core may be confusing at first glance. However, it should be clearly noted here, that the similar bi-exponential fluorescence decays in algal PSI core, with lifetimes ~7 and 23–24 ps both for open and closed RCs (but with varying relative amplitudes in the two states), was modeled differently than in the case of cyanobacterial PSI. Due to the low amount of red Chls and a minor contribution of the bulk/red equilibration in algal PSI core, the bi-exponential decay was modeled assuming reversibility of the primary charge separation:

10 $$({\text{Ant/RC}})^{*} \mathop \leftarrow \limits^{{\tau_{ - 1} }} \mathop \to \limits^{{\tau_{1} }} {\text{S}}_{1} \mathop \to \limits^{{\tau_{\text{irr}} }} {\text{S}}_{2}$$

where S_1_ is the primary charge separated state, and S_2_ is secondary charge separated state (in the case of open RC) or an uncertain state, maybe ground state recovered after charge recombination depicted by the molecular lifetime *τ*_irr_ (in the case of closed RC). Since the excitation energy transfer from bulk to red Chls is evident in the case of cyanobacterial PSI and model (10) ignores such a reaction, we could not have applied this model to cyanobacterial PSI. Instead, we connected the model (10) with the one shown in Figs. 3 and 5:

11 $$\text{Red}\mathop \leftarrow \limits^{{\tau_{2} }} \mathop \to \limits^{{\tau_{3} }} {\text{Bulk}}\mathop \leftarrow \limits^{{\tau_{ - 1} }} \mathop \to \limits^{{\tau_{1} }} {\text{S}}_{1} \mathop \to \limits^{{\tau_{\text{irr}} }} {\text{S}}_{2}$$

However, probably due to too many reactions occurring on the same time scale, we were not able to obtain physically interpretable results from such a model. Therefore, the simplified model, describing the photochemical quenching by one molecular lifetime (*τ*_1_), was used for our results:

12 $$\text{Red}\mathop \leftarrow \limits^{{\tau_{2} }} \mathop \to \limits^{{\tau_{3} }} {\text{Bulk}}\mathop \to \limits^{{\tau_{1} }} {\text{S}}$$

The same approach to target analysis of fluorescence decays in various cyanobacterial PSI preparations was used recently in van Stokkum et al. (2013). Obviously, this does not mean that our results exclude the possibility of reversible charge separation in cyanobacterial PSI. Moreover, a quite precise comparison of the primary charge separation rate between cyanobacterial PSI monomers and algal PSI cores can be done based on our results, and those published in Giera et al. (2014). Assuming that the excitation decay in algal PSI core is described by the simplified model (Eq. 12) and taking into account the fact that energy equilibration between bulk and red chlorophylls has a negligible impact on the excitation dynamics in this system, we can say that the overall fluorescence decay in algal PSI core would be described by only one molecular lifetime (*τ*_1_). Obviously, we can expect that this lifetime modeled in target analysis would be almost the same as an average fluorescence lifetime calculated based on global analysis. Therefore, molecular lifetimes (*τ*_1_) obtained here for cyanobacterial PSI monomers (12.8 and 16.1 ps, for open and closed RCs, respectively, Table 1) should be compared with the average lifetimes reported in Giera et al. (2014; 13.8 and 17.4 ps; Table 1) rather than with molecular lifetimes obtained in Giera et al. (2010; 8.9 and 11.4 ps; Table 1) based on a different model. Such a comparison leads to the conclusion that the rate of trapping of bulk excitations in RC is almost the same in cyanobacterial PSI and in algal PSI core.

Another interesting issue associated with molecular lifetimes is the acceleration of photochemical quenching to ~10–11 ps in iPSI compared to ~15–16 ps for closed PSI in solution (Table 1). Interestingly, the molecular lifetime of photochemical quenching in iPSI is comparable with the molecular lifetime of the forward electron transfer rate obtained in the reversible charge separation model for algal PSI core with closed RC (Giera et al. 2010; 11.4 ps; Table 1). It should be emphasized here that in closed PSI (with P700 permanently oxidized to P700^+^) the primary charge separation likely leads to formation of the state A^+^A_0_^−^ (Holzwarth et al. 2006; Giera et al. 2010). If the reverse reaction in the primary charge separation step in algal PSI core (closed RC), A^+^A_0_^−^ → (Ant/RC)*, was blocked, then the overall fluorescence decay in this system would be described directly by the forward reaction rate equal to ~11 ps. Therefore, we can hypothesize here that the observed acceleration of photochemical quenching in the iPSI is the effect of blocking the reverse reaction in the primary charge separation (if we only assume the reversibility of the primary charge separation in cyanobacterial PSI, Bulk ⇆ A^+^A_0_^−^). This blockage of the reverse reaction could be the result of very fast re-reduction of A^+^ by electron from FTO or injection of electron from A_0_^−^ to FTO. Similar effects are well documented for dyes on conducting surfaces (see for instance Tachibana et al. 1996; Horvath et al. 2015; Kleimeier et al. 2015; Ashford et al. 2015). An alternative explanation of the fluorescence decay acceleration in iPSI may be strong aggregation of proteins on FTO caused by low concentration of detergent and tight packing. Fluorescence quenching is well documented for aggregates of LHC II particles (Vasil’ev et al. 1997; Andreeva et al. 2009) or chlorophylls in solution (Shi et al. 2013).

The numbers of red Chls estimated by us from the target analysis for monomeric and trimeric PSI in solution (Table 1) are strikingly similar to those published previously (Gobets et al. 2003): 3 red Chls per monomer for monomeric PSI and 4 red Chls per monomer for trimeric PSI. The latter number fits perfectly the idea that in PSI trimers extra red Chls are formed at the interfaces of interacting monomers—one for each of the three interfaces. This consistency of our results with previous studies gave us confidence in the way of how we estimated the number of red Chls and prompted us to perform similar estimations for PSI deposited on FTO. Surprisingly, whereas trimers deposited on the substrate preserved the number of 4 red Chls per monomer, the number of long-wavelength forms increased from 3 to 6 in the case of monomeric PSI. We speculate that each of the adsorbed and dried monomeric PSI strongly interacts with the neighboring monomers and with the substrate due to low amount of detergent (see Materials and methods) and dense packing and thus small distance between monomers, yielding even more extra red Chls at the interfaces than we observe at the interfaces of the monomers forming the trimeric PSI. The redshift of the extra red Chls is likely caused by charge transfer states mixed with excitonic states of peripheral Chls not well protected by the protein. Since trimeric PSI deposited on FTO preserves their number of red Chls in solution, we propose that the monomeric protein surfaces susceptible to formation of red Chls are limited to the region of interaction within a trimer which is apparently protected against increased interaction after deposition on FTO. Careful analysis of cofactors’ positions in PSI from *Synechococcus elongatus*, a cyanobacterium for which the crystallographic structure was solved (Jordan et al. 2001) reveals that there are 12 Chls located in this region close to the protein surface. According to the notation in Jordan et al. (2001), these Chls are: CL11120, CL11121, CL11134, CL11208, CL11209, CL11212, CL11217, CL11401, CL11402, CL11501, CL11601, CL11801. Appearance of extra red Chls (per monomer) in trimeric PSI from various cyanobacteria as a result of trimerization is well documented (Gobets et al. 2001; Karapetyan 2004; Karapetyan et al. 2014). Also aggregation of LHC II leads to appearance of a few long-wavelength Chls (Vasil’ev et al. 1997). These observations are in line with our result of increased number of red Chls in monomeric iPSI forming aggregates on FTO.

Interestingly, the number of red Chls seems not to influence significantly the overall fluorescence decay. In solution, extra red Chls in trimers do not slow down excitation trapping compared to monomeric PSI (Fig. 2; Table 1). Rather the opposite tendency is observed. This may be explained by competition of two effects. In the monomers, there are less red Chls, but in a fraction of monomers a “slow” trapping occurs due to energetic disorder. In the trimers, there are more red Chls which slow down trapping but the connection between the three monomeric PSI complexes accelerates trapping because an exciton that arrives at a “slow” monomer can escape to a “fast” monomer. Similarly, monomeric PSI on FTO despite having six red Chls (two more than trimeric PSI on FTO) show slightly faster fluorescence decay than trimeric PSI (Fig. 2b; Table 1). Like above, we propose that the connections between monomers in the case of monomeric iPSI lead to fast energy transfer between them thus overcoming the slowing effect of the increased number of red Chls by the good connection between energetically disordered “slow” and “fast” monomers.

Interestingly, adsorption of PSI monomers and trimers affects not only the number of red Chls (in the case of monomeric PSI), but also the interaction between bulk and red Chls: both forward and backward energy transfer rates between these two pools decrease after immobilization (both for monomeric and trimeric PSI) although the free energy gap between them is strongly affected only for monomeric iPSI (−17 meV for iPSI instead of −27 meV for PSI in solution; Table 1). Thus, we conclude that immobilization and drying of cyanobacterial PSI on FTO only slightly modifies interactions between Chls in the antenna system.

Based on our approach, it is difficult to conclude on the nature and exact localization of the red Chls. However, our results are consistent with the idea that the additional, fourth red Chl in each monomer forming the trimeric PSI is at the very periphery of a monomer, in its contact region with another monomer. In our modeling (Figs. 3, 5), we also assumed that red Chls do not transfer excitation energy directly to RC, but always via bulk Chls, meaning that they are not located in the very vicinity or inside the RC. Assuming that red Chls are formed as charge transfer states mixed with excitonic states of interacting Chl dimers of trimers (Romero et al. 2008; Novoderezhkin et al. 2016), we may only propose that in the case of monomeric PSI in solution their effective number of three red Chls may be a result of strong interactions within either only one trimer, two or more dimers, or a combination of trimer(s) or dimer(s).

In conclusion, we state that first steps of energy and electron transfer in monomeric and trimeric cyanobacterial PSI are well preserved and even slightly accelerated after removal of water solvent and after dense protein packing on the flat surface of FTO conducting glass. The trimeric PSI is less susceptible to modifications of Chl–Chl interaction after immobilization. These observations further confirm the usefulness of cyanobacterial PSI in biophotovoltaic applications.
